# The Surgical Aspect of Cholelithiasis

**Published:** 1905-09

**Authors:** James Swain

**Affiliations:** Professor of Surgery at University College, Bristol; Surgeon to the Bristol Royal Infirmary


### THE SURGICAL ASPECT OF CHOLELITHIASIS.

BY

James Swain, M.S., M.D. Lond., F.R.C.S.,

*Professor of Surgery at University College, Bristol;*
*Surgeon to the Bristol Royal Infirmary.*

### I.

Although Petit, about 1743, suggested with wonderful foresight that gallstones should be removed by operation, it was not till within the last twenty years that the surgery of the gall-bladder and its ducts made any appreciable advance. It is not, therefore, surprising that the knowledge of what can be effected by timely interference is scarcely as widely known as the subject deserves.

In looking over my notes of operations for cholelithiasis, a consideration of the difficulties in diagnosis and the question of operative treatment seemed an efficient means of drawing attention to the subject.

A gall-bladder may be distended by mucus (catarrhal cholecystitis), pus (suppurative cholecystitis, empyema), or bile. The enlargement is perhaps most commonly mistaken for a movable kidney. The enlarged gall-bladder lies immediately under the parietes, and unless adhesions are present can be moved laterally, and ascends and descends with the liver during respiration. It always has, however, a fixed point at the liver about opposite the tenth costal cartilage, and if an attempt is made to push it into the flank it springs back into its normal position. The movable kidney is more deeply situated, it recedes into the loin on pressure with a characteristic slip, and when reduced stays in position. If associated with pain, this is referred downwards along the ureter to the groin. The kidney is covered by the colon, but too much stress should not be placed on the existence of resonance due to this fact, as an enlarged gall-bladder may also be overlapped by the colon, owing to the presence of adhesions.

Extensive inflammatory adhesions around the gall-bladder may cause some confusion with tumours of the pylorus, colon, or omentum, but if an attempt be made to hold the tumour after it has descended on deep inspiration, it will be found that it is impossible to keep the gall-bladder down during expiration, whereas the other tumours referred to can be so held unless they have formed adhesions to the liver. The diagnosis is perhaps of less importance in these cases, as the presence of a tumour would probably be considered a sufficient justification for operation.

In acute cholecystitis the existence of tenderness and fever may also be of service in the diagnosis, but it is most important to bear in mind that so dangerous a condition as suppurative cholecystitis can exist in a chronically thickened gall-bladder without any perceptible tumour at all.

Pain is one of the most characteristic symptoms of gallstone disease, but it varies so much in severity and locality that errors

15

in diagnosis often occur. The pain of catarrhal cholecystitis may be slight and may pass off in a few hours, but the pain of a suppurative cholecystitis is often very severe, especially if it is associated, as it frequently is, with inflammation of the adjacent peritoneum. The tenderness is so great in these cases that a satisfactory examination is often not possible.

So long as the gallstones are confined to the gall-bladder the pain is chiefly felt in the right hypochondrium, and radiates to the breast and back, but if the stones are forced into the cystic or common duct the pain and tenderness are frequently most severe in the epigastrium. In these latter cases the pain is of a very violent character as obstruction is added to inflammation.

The pain thus excited has frequently led to an erroneous diagnosis of gastric ulcer, and I feel sure that most cases of so-called gastric cramp are really due to gallstone colic. The pain of gallstones comes on generally without any exciting cause, and is not influenced by food, as in the case of gastric ulcer. A test meal will show the presence of hyperchlorhydria in the latter case, and such symptoms as hæmatemesis or jaundice will, if they occur, aid in the differential diagnosis of ulcer or gallstones respectively.

Gallstone colic is often so severe and sudden that it has been mistaken for intestinal obstruction or some form of peritonitis, such as that due to ruptured gastric ulcer, appendicitis, &c. In these cases we have the symptoms—pain, collapse, vomiting—which have been grouped under the term "peritonism," and which may be said to be common to all acute crises in the abdomen. A right diagnosis may be made only after watching the case for a time, but the presence of well-marked tenderness in the right hypochondrium should put us on our guard as to the probable cause. In peritonitis the abdomen, in the early stage, is retracted and rigid, and the respiration is of the costal type; but in gallstone colic the diaphragm usually continues to act.

A rapid and fatal form of peritonitis does however occur in acute phlegmonous inflammation of the gall-bladder, leading to gangrene. The symptoms are severe and unmistakable, and such a peritonitis occurring in the right hypochondrium demands immediate operation.

A less severe, though serious, peritonitis also occurs in association with suppurative cholecystitis, and the existence of pain, fever, and tenderness accounts for its confusion with appendicular peritonitis. The difficulty is increased by the fact that the appendix frequently runs in an upward direction, but in cholecystitis the tumour (if it exists) and the point of maximum tenderness are closer to the costal margin than in appendicitis. Catarrhal cholecystitis tends to recur, and thus a resemblance to appendicitis occurs; but here again the situation of the point of greatest tenderness will elucidate the diagnosis.

When a gallstone enters the common duct the patient frequently suffers from ague-like paroxysms, in which an initial rigor is followed by a rapid elevation of temperature—104° to 105° F.—which falls abruptly to normal in a few hours. These attacks may occur several times a day, but their marked irregularity at once distinguishes them from malarial fever. A remittent or intermittent jaundice often accompanies these attacks, but it is not sufficiently understood that jaundice is not a necessary accompaniment of stone in the choledochus. I have removed stones from the common duct in which no jaundice occurred, but in these cases bile pigments were present at times in the urine.

If jaundice occurs we have to differentiate between the presence of a calculus in the choledochus and jaundice produced by pressure on the duct from tumours (malignant disease) or inflammatory conditions of the pancreas (chronic pancreatitis). If the jaundice has set in suddenly, accompanied by colicky pains, the diagnosis is in favour of stone, and if there is a distinct history of remission of the jaundice there is an almost absolute certainty that a stone in the choledochus is the cause. On the other hand, a steady increase in the jaundice without remission points to some external cause (tumour, pancreatitis), and especially if the jaundice is very deep and unaccompanied by fever or pain. The condition of the gall-bladder in these circumstances is worth noting. Owing to previous inflammatory attacks in association with calculi the walls of the gall-bladder are thickened and incapable of distension. This does not obtain in jaundice due to pressure from without, so that deep jaundice in association with a distended gall-bladder is in favour of malignant disease. I have, however, met with several exceptions to this. Cachexia, and the enlarged gland ("Virchow's gland") at the base of the neck which occur in cases of malignant disease, appear too late to be of value in the diagnosis, and I have successfully removed stones from the common duct in a patient in whom there was an appearance of cachexia which had led to a diagnosis of cancer. In fact, the differential diagnosis of these three conditions—stone in the common duct, malignant disease, and chronic pancreatitis—is beset with considerable difficulty, so that it is often necessary to advocate an exploratory operation for the purpose of clearing up the matter.

When the disease has lasted for a considerable time, malignant disease can be fairly easily diagnosed or excluded; but that still leaves us with the uncertainty in many cases as to whether we are dealing with stone in the common duct or chronic pancreatitis.

Mr. Cammidge[1] has sought to dispel the difficulties just referred to by an examination of the urine; but with the assistance of Mr. Taylor, the dispenser at the Royal Infirmary, I have tried the tests suggested in several cases, and have not found them of any practical value.

So, too, as I showed some years ago,[2] the X-rays are not likely to be helpful in the diagnosis of cholelithiasis, as the shadow cast by gallstones is very slight.

It may be helpful to tabulate some of the chief points which are worth noting in association with the gall-bladder and its ducts.

1. A gall-bladder which has been repeatedly inflamed may not be capable of distension, so that a suppurative cholecystitis may exist without a palpable swelling.

[1] *Brit. M. J.*, 1904, i. 776.

[2] "The Effect of the Röntgen Rays in Calculi," *Bristol M.-Chir. J.*, 1897, xv. i.

2. A painless enlargement of the gall-bladder without jaundice suggests catarrhal cholecystitis.

3. A painful enlargement of the gall-bladder without jaundice suggests an empyema of the gall-bladder.

4. A painless tumour of the gall-bladder with jaundice is in favour of an obstruction of the common duct by a tumour (malignant).

5. Remittent jaundice, generally without an enlarged gall-bladder, accompanied by chills and pain, occurs with stone in the common duct.

6. A hard, nodular, painful tumour of the gall-bladder, with or without jaundice, denotes carcinoma of the gall-bladder.

7. Jaundice is a less frequent symptom in cholelithiasis than is usually supposed. Kehr says it is absent in 80 per cent. of cases.

### II.

Though gallstone disease is extremely common, we find that only a small proportion of people suffer actively from it. Even in those who have definite attacks of gallstone colic the mischief frequently subsides under appropriate forms of medical treatment which it is not my intention to discuss; but in these cases it is the inflammatory phenomena only which subside—the gallstones remain in most cases. Quiescent stones may be practically ignored, though we must remember that ulceration and carcinoma may develop from the presence of such concretions.

Patients so quickly recover after their attacks of gallstone colic that we can scarcely wonder at the tendency among both the profession and the laity to delay operation until some definite pathological lesion has been set up; but when one sees the late results of gallstones—such as empyema, cholangitis, perforation and impaction of stones in the choledochus, all of which are associated with a high degree of mortality—the surgeon is justified in his anxiety to operate while the gallstones are in the gall-bladder, when the mortality does not exceed one per cent. As these facts become more widely known it is probable that the present custom of delay may give place to an earlier prophylactic operation after the manner of its comparatively recent adoption in appendicitis. More than half of my cases of operations for gallstones have been performed while the stones were in the gall-bladder, and not one of these simple cases died. Against this satisfactory result we have the fact that operation for stones in the common duct (choledochotomy) has a mortality of 10 per cent. or more.

By this suggestion of early operation it is not to be understood that I am advocating operation in every case. Such an idea is worthy of as great condemnation as the habit of delay to which I have referred. It will therefore be useful to first consider those cases in which operation is undesirable.

In mild and infrequent attacks of cholecystitis the patient would probably be unwilling to consider the question of operation, and in such cases it cannot be considered absolutely necessary, even though we know that many of the mild cases slowly progress towards serious pathological disease. If, however, the gall-bladder is enlarged I think an operation should be recommended, though the case may be otherwise of a mild type (*v. infra*).

Even with frequent and severe attacks of colic associated with the passage of small calculi an operation is unnecessary so long as the stones continue to pass. Operation is indicated if the frequent attacks of colic cease to be attended with the expulsion of calculi.

In acute obstruction of the common duct operations should not be performed, as such cases not infrequently get well under expectant treatment. If, however, the jaundice persists for some five or six weeks, or if symptoms of cholangitis—as shown by rigors, intermittent fever, enlargement of the liver, &c.—supervene, operation is desirable; for under such circumstances the risk of operation is far less than that of expectant treatment.

In old patients, and those suffering from organic disease of the heart, lungs, or kidneys, operation is rarely justifiable, and in any case should only be undertaken on account of the development of some grave condition associated with the gallstones.

On the other hand, operation should be advocated in cases of severe and frequent colic, incapacitating the patient from the necessary duties of life.

In all cases of enlarged gall-bladder, whether painful or not, and whether accompanied by jaundice or not, operation is the safest course to pursue. This will include some of the catarrhal and suppurative affections of the gall-bladder; but it must also be remembered that suppurative cholecystitis may occur in a contracted gall-bladder without causing any palpable tumour.

The occurrence of acute diffuse peritonitis in the neighbourhood of the gall-bladder may be associated with acute phlegmonous inflammation of the gall-bladder, or perforation of the gall-bladder or ducts. Such conditions are extremely grave, and with any indication of their existence operation is imperative.

Painful adhesions between the gall-bladder and the stomach or intestines should be released by operative means.

I have already said that a chronic obstruction of the common duct — especially if associated with cholangitis or symptoms of liver abscess — should be treated by operation, and some observers state that they have had success after operation in cases where the patients have been addicted to morphia in consequence of gallstone colic.

The pathological lesions in many of the cases referred to are so serious that operation is necessarily attended with a high mortality. This mortality, though the operations are often tedious and difficult, is largely due to delay, and might often be avoided by an earlier resort to surgical interference. Each case must necessarily be considered on its merits; but where medical treatment has failed, or where the more serious symptoms are threatening, we may be sure that operative procedures are safer than a continuation of expectant treatment, and our aim should be, if possible, to operate before the stones have left the gall-bladder and become impacted in the ducts.

